# A Murine Model of *falciparum*-Malaria by *In Vivo* Selection of Competent Strains in Non-Myelodepleted Mice Engrafted with Human Erythrocytes

**DOI:** 10.1371/journal.pone.0002252

**Published:** 2008-05-21

**Authors:** Iñigo Angulo-Barturen, María Belén Jiménez-Díaz, Teresa Mulet, Joaquín Rullas, Esperanza Herreros, Santiago Ferrer, Elena Jiménez, Alfonso Mendoza, Javier Regadera, Philip J. Rosenthal, Ian Bathurst, David L. Pompliano, Federico Gómez de las Heras, Domingo Gargallo-Viola

**Affiliations:** 1 Diseases of the Developing World, Infectious Diseases-Centre for Excellence in Drug Discovery (ID CEDD), GlaxoSmithKline, Tres Cantos, Madrid, Spain; 2 Department of Anatomy, Histology and Neuroscience, Faculty of Medicine, Universidad Autónoma de Madrid, Madrid, Spain; 3 Department of Medicine, San Francisco General Hospital, University of California San Francisco, San Francisco, California, United States of America; 4 Drug Discovery and Technology, Medicines for Malaria Venture (MMV), Geneva, Switzerland; 5 Infectious Diseases-Centre for Excellence in Drug Discovery (ID CEDD), GlaxoSmithKline, Collegeville, Pennsylvania, United States of America; Swiss Tropical Institute, Switzerland

## Abstract

To counter the global threat caused by *Plasmodium falciparum* malaria, new drugs and vaccines are urgently needed. However, there are no practical animal models because *P. falciparum* infects human erythrocytes almost exclusively. Here we describe a reliable *falciparum* murine model of malaria by generating strains of *P. falciparum in vivo* that can infect immunodeficient mice engrafted with human erythrocytes. We infected NOD*^scid/β2m−/−^* mice engrafted with human erythrocytes with *P. falciparum* obtained from *in vitro* cultures. After apparent clearance, we obtained isolates of *P. falciparum* able to grow in peripheral blood of engrafted NOD*^scid/β2m−/−^* mice. Of the isolates obtained, we expanded *in vivo* and established the isolate *Pf*3D7^0087/N9^ as a reference strain for model development. *Pf*3D7^0087/N9^ caused productive persistent infections in 100% of engrafted mice infected intravenously. The infection caused a relative anemia due to selective elimination of human erythrocytes by a mechanism dependent on parasite density in peripheral blood. Using this model, we implemented and validated a reproducible assay of antimalarial activity useful for drug discovery. Thus, our results demonstrate that *P. falciparum* contains clones able to grow reproducibly in mice engrafted with human erythrocytes without the use of myeloablative methods.

## Introduction

The erythrocytic stages of the most virulent human malaria parasite, *P. falciparum*, are responsible for hundreds of millions of illnesses and over one million deaths every year [1]. Due to its exquisite specificity, only human beings and a small number of non-human primates are susceptible to infection by the erythrocytic stages of *P. falciparum* [2]. The lack of available animals, high costs and overt ethical problems have precluded the widespread use of primates in malaria research. As an alternative, rodent or avian plasmodial species non pathogenic for humans have been used as surrogates of *P. falciparum* [3]. However, in spite of their value, there are significant biological differences between these species and the human parasite [4], [5].

A reliable murine model of *P. falciparum* malaria would be a valuable research tool, particularly in drug discovery [6]. At least, such a model should guarantee that the parasite grows in a predictable way in peripheral blood of mice having circulating human erythrocytes (hE) in bloodstream, the physiologically relevant compartment. This requires the availability of susceptible hE, the competence of *P. falciparum* to grow using the nutrients available and the ability of *P. falciparum* to overcome the innate immune system of mice engrafted with human erythrocytes (humanized mice, HM). So far, hE have been successfully grafted into *nude* or *scid* immunodeficient mice upon intraperitoneal injection using [7]–[11] or not [12] immunosuppressive treatments. However, obtaining sustained infections with *P. falciparum* in HM has only been possible by depleting *in vivo* tissular macrophages and neutrophils with dichloromethylene diphosphonate-containing liposomes plus anti-neutrophil NIMP-R14 mAb [8]–[11], [13]. Unfortunately, the different versions of this model require intraperitoneal infection, have a high rate of failure [8], [9], [13] or show limited reproducibility of infection outcome [10], [11], display variable kinetics of parasitemia, and use toxic reagents, which might interact in unknown ways with antimalarials or effector cells. These shortcomings have limited its use in drug discovery [3] and vaccine development, despite several standard antimalarials [9] and antibodies [13]–[15] were shown to have activity in this model.

In order to develop a *falciparum* murine model useful for drug discovery, we hypothesized that laboratory or clinical strains of *P. falciparum* should contain clones able to survive and be expanded *in vivo* in non-myelodepleted HM. Supporting this hypothesis, firstly, *P. falciparum* 3D7 adapted to grow *in vitro* in cultures containing murine ascites could replicate intraperitoneally in NOD*^scid^* HM boosted daily with hE even though parasitemias in peripheral blood were erratic and parasites were readily cleared from mice [12], [16]. Secondly, phagocyte-depleted HM are metabolically permissive for *P. falciparum* laboratory and clinical isolates [9], [11]. Finally, *P. falciparum* can evade host's innate immune system in natural infections by antigenic variation through expression of alternative genes of multigene families [17], [18]. Here we describe the first murine model of *falciparum* malaria after intravenous infection of non-myelodepleted humanized NOD*^scid/β2m−/−^* mice with *Pf*3D7^0087/N9^, to our knowledge the first strain of *P. falciparum* generated specifically for a murine model, and demonstrate its value as a reliable tool for drug discovery.

## Results

### Selection of the immunodeficient murine strain

**Figure 1:**
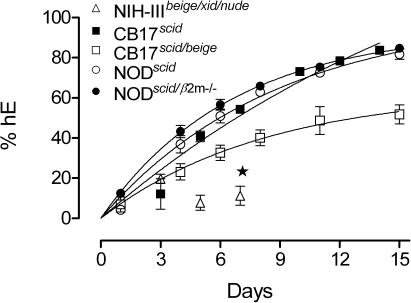
Selection of immunodeficient mice. The percentage of hE (TER-119^−^ or human glycophorin A^+^ cells) in peripheral blood of NIH-III*^beige/xid/nude^*, CB17*^scid^*, CB17*^scid/beige^*, NOD*^scid^* and NOD*^scid/β2m−/−^* murine strains upon daily intraperitoneal injection of hE is shown. The regression curves for fitting to an exponential association equation are shown for CB17*^scid^*, CB17*^scid/beige^*, NOD*^scid^* and NOD*^scid/β2m−/−^*. The strain NIH-III*^beige/xid/nude^* was discarded from the study at the point indicated (★). Data are the mean±SE of n = 4 mice per data point.

The innate immunity of immunodeficient *scid* and *nude* mice is able to reject xenotranplants [19] and eradicate parasites [20]. We tested five immunodeficient murine strains for engraftment of A+ hE: NIH-III*^beige/xid/nude^*, CB17*^scid^*, NOD*^scid^*, which have already been shown to be able to accept hE [7], [9], [12]; CB17*^scid/beige^*, which has no NK activity [21], [22] and NOD*^scid/β2m−/−^*, which is one of the best acceptors of human xenotransplants [23], [24]. We administered 1 ml of hE A+ 50% hematocrit 25% human serum 3.1 mM hypoxanthine in RPMI 1640 daily by i.p. to maximize the volume of suspension injected and obtain a sustained delivery of erythrocytes to the vascular system, likely through lymphatic drainage [12]. The hE suspension contained human serum and hypoxanthine because human serum increases the half life of engrafted hE [7] and both components might enhance the growth of *P. falciparum*. We injected four mice of each strain daily with hE and measured the degree of engraftment over 15 days (Fig. 1). Consistently with previous reports [9], [10], NIH-III*^beige/xid/nude^* mice were almost refractory to engraftment. Conversely, CB17*^scid/beige^*, CB17*^scid^*, NOD*^scid^*, and NOD*^scid/β2m−/−^* mice were engrafted fitting a one phase exponential association equation, with CB17*^scid^*, NOD*^scid^* and NOD*^scid/β2m−/−^* showing the best engraftment (Fig. 1). Finally, we selected NOD background mice for further experiments because those mice have defects in serum complement, NK cell activity and macrophage activation whereas these components are intact in CB17 mice [25].

### Selection of a competent *P. falciparum* 3D7 strain

**Figure 2:**
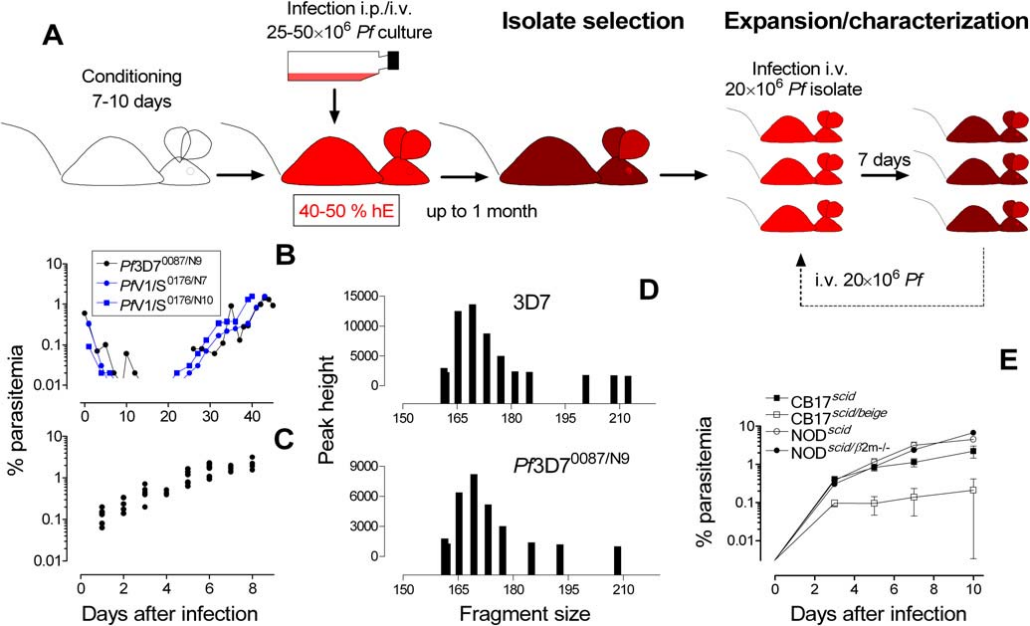
Optimized selection and characterization of competent *P. falciparum* isolates. (A) A cohort C1 of mice is conditioned for 7–10 days with daily i.p. injections of hE. Then, the mice are infected by i.p. or i.v. route with 25·10^6^ or 50·10^6^ parasitized erythrocytes from *in vitro* cultures. Parasitemia in peripheral blood is followed up to 1 month after infection or until about 1% of parasitemia is achieved (see point B). Mice having productive infections are used as donors to infect by i.v. route a cohort C2 of conditioned mice. After 1 week of infection, the mice with the highest parasitemia is selected as donor for next *in vivo* passage. After ten passages showing a stable kinetics of growth (see point C), a final expansion step is performed to establish a standard *P. falciparum* strain, which is frozen and used in experiments. (B). Course of parasitemia in peripheral blood from the mice that originated the competent isolates *Pf*3D7^0087/N9^ (i.p. infection), *Pf*V1/S^0176/N7^ (i.p. infection) and *Pf*V1/S^0176/N10^ (i.v. infection). (C) Dot plot analysis of the kinetic stability of the isolate *Pf*3D7^0087/N9^ growth during sequential i.v. infections with 20·10^6^ *P. falciparum*-infected erythrocytes. Stability in 10 sequential i.v. infections was used as the criterion for establishing the reference standard *Pf*3D7^0087/N9^ strain. Each data point is the mean of three mice per *in vivo* passage for ten consecutive passages. (D) Microsatellite *Pf*RRM of the *P. falciparum* 3D7 and *Pf*3D7^0087/N9^ strains. (E) Growth of *Pf*3D7^0087/N9^ in different strains of HM after i.v. infection with 20·10^6^ *Pf*3D7^0087/N9^ parasites. Data are the mean±SE of four mice·group^−1^.

We hypothesized that some clones of *P. falciparum* could survive in peripheral blood of HM without using chemical phagocyte depletion. Although cultured *in vitro* for decades, we selected *P. falciparum* 3D7 because it was the one used by Moore et al [12], it is well characterized and still is the most widely used for *in vitro* drug testing. Then, we infected 10 NOD*^scid^* and NOD*^scid/β2m−/−^* HM having 40–50% of chimerism (7–9 days after starting i.p. injections of hE) with i.p. injections of 1 ml 50% hematocrit with approximately 2% of parasitemia because the i.p. route was the only route for which successful infections were obtained previously [9], [12], [13], [26] (Fig. 2, A). Similarly to Moore et al [12], after some initial replication, all NOD*^scid^* mice cleared parasites from peripheral blood. However, after apparent clearance, two NOD*^scid/β2m−/−^* mice showed productive infections at days 26 and 30 after parasite inoculation (Fig. 2, B). The frequency of successful infections in the experiments performed (3 different experiments, n = 5, n = 10 and n = 12, respectively) was about 10–20%. Therefore, *P. falciparum* 3D7 growing *in vitro* contained parasites able to survive in NOD*^scid/β2m−/−^* HM not treated with myelosuppressive drugs. This was not restricted to *Plasmodium* 3D7 and the i.p. route of infection because after i.p. or i.v. infection of NOD*^scid/β2m−/−^* HM with *P. falciparum* V1/S (n = 10 mice), a multiresistant clinical isolate adapted to *in vitro* culture, the percentage of success was 100%. This confirmed that other strains contain parasites competent to grow in NOD*^scid/β2m−/−^* HM (Fig. 2, B).

Next, we demonstrated that surviving parasites could be transmitted and expanded *in vivo* in NOD*^scid/β2m−/−^* HM, which we selected for further experiments. We infected i.p. three HM per donor with ∼25·10^6^ parasites obtained from two cohort 1 mice having ≥1% parasitemia in peripheral blood (named isolates *Pf*3D7^0087/N5^ and *Pf*3D7^0087/N9^). All mice were infected and showed parasitemias between 0.1 and 8% for more than one month, similarly to successful infections obtained by Moreno et al [9]. As we obtained the same results in four sequential infections using the first mice of each passage achieving parasitemias ≥1% as donor for the next cohort, we concluded that both isolates could be transmitted *in vivo*. The erratic parasitemias observed in mice could be due to the growth of *P. falciparum* 3D7 that we observed in the peritoneum of HM, as described in other models [12], [26]. Interestingly, all mice inoculated i.v. with 20×10^6^ *Pf*3D7^0087/N5^- or *Pf*3D7^0087/N9^-parasites showed productive infections. In all cases, we obtained a reproducible exponential growth and achieved parasitemias above 1% one week after infection for ten consecutive i.v. passages *in vivo* (Fig. 2, C). Thus, we expanded *Pf*3D7^0087/N5^ and *Pf*3D7^0087/N9^ isolates infecting 14 and 40 mice by i.v. route with 20·10^6^ parasites, respectively. The parasites grew exponentially in all HM mice and we froze parasites one week after infection for establishing parasite stock reference collections for standardization. The isolates *Pf*3D7^0087/N5^ and *Pf*3D7^0087/N9^ had parasitemias at freezing of 2.7±0.15 and 2.28±0.14%, respectively.

Finally, we selected the strain *Pf*3D7^0087/N9^ for full model characterization. This strain produced a characteristic pattern of nine bands after microsatellite *Pf*RRM fingerprinting [27] whereas the original *P. falciparum* 3D7 showed eleven DNA fragments, eight of them identical to *Pf*3D7^0087/N9^ (Fig. 2, D). Interestingly, *Pf*3D7^0087/N9^ grew *in vitro* as the parental *P. falciparum* 3D7 (duplication time ∼1 day), either cultured from frozen stocks or when taken directly from the peripheral blood of infected mice, and maintained the parental pattern of susceptibility to control antimalarial compounds (Table 1). In addition, *Pf*3D7^0087/N9^ was able to grow after i.v. infection in murine strains successfully engrafted with hE (CB17*^scid^* and NOD*^scid^*) (Fig. 2, E). Hence, the procedure described above led to selection of a variant of *P. falciparum* 3D7 competent to grow reproducibly in peripheral blood of HM.

**Table 1:** Comparison of the activity of selected antimalarials against *P. falciparum* 3D7 or *Pf*3D7^0087/N9^ 1.

Compound	*P. falcipaum* strain	
	3D7	*Pf*3D7^0087/N9^
Artesunate	1,1±0,4^2^	1,3±0,4
Chloroquine	16,2±0,5	11,1±3,6
Artemisinin	4,1±1,5	4,9±0,7
Pyrimethamine	4,6±0,8	3,9±0,8
Atovaquone	0,2±0,07	0,3±0,09

1 The activity of antimalarials is expressed as the concentration in ng·ml^−1^ that inhibits by 50% the incorporation of [^3^H]-hypoxanthine (IC_50_).

2 Differences between IC_50_ were not statistically significant in any case.

### Long-term chimerization

**Figure 3:**
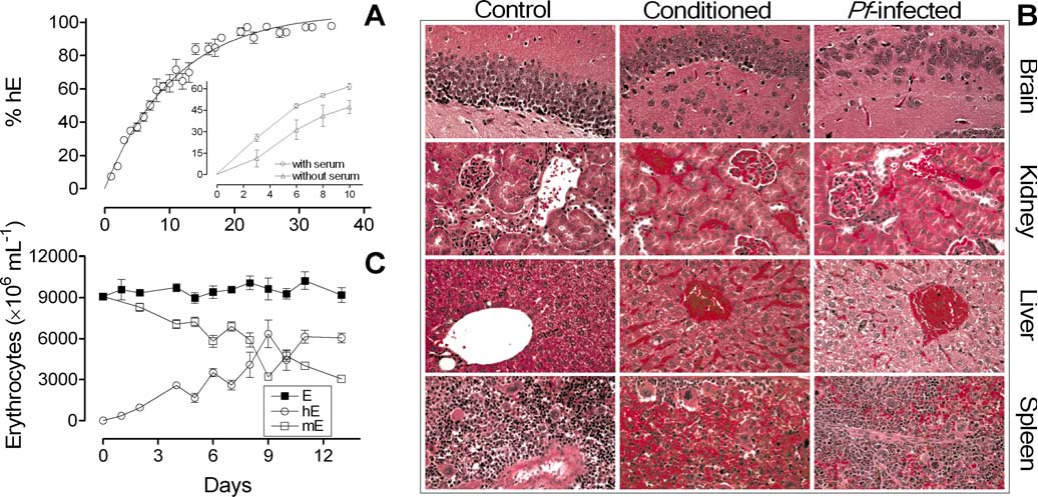
Engraftment of hE in NOD*^scid/β2m−/−^*. (A) Long term kinetics of engraftment of NOD*^scid β2m−/−^* mice with hE. Data are the mean percentage±SE of hE (TER-119^−^ or human glycophorin-A^+^ mouse) erythrocytes pooled from 142 mice. The inset plot shows the effect of human serum deprivation from daily-inoculated hE on engraftment of hE in peripheral blood of NOD*^scid β2m−/−^* (n = 3 mice·group^−1^). Data shown are from a representative experiment out of three. (B) Histological analysis of brain, kidney, liver and spleen of NOD*^scid/β2m−/−^* control, conditioned with hE and conditioned mice infected with *P. falciparum* (day 8 after infection). Non-infected mice conditioned with hE showed a marked vascular congestion compared to control mice. Infected mice, showed decreased vascular congestion, increased numbers of myelomonocytic cells and enhanced phagocytic activity in the spleen (×600 magnification). (C) Concentration of erythrocytes, hE and mE in peripheral blood of NOD*^scid β2m−/−^* during conditioning before i.v. infection with *P. falciparum*. Data are the mean±SE of n = 21 mice.

Next, we analyzed the effects of long-term daily injections of hE in NOD*^scid/β2m−/−^* mice. In practice, 100% of mice were successfully grafted with hE. The acquisition of chimerism fitted a one phase exponential association equation (R^2^ = 0.75, n = 162 mice, t_1/2_ = 7.5±0.6 days) reaching levels of >90% hE by day 25 (Fig. 3, A) irrespective of their ABO or Rh group (not shown). The elimination of human serum from erythrocyte suspensions slightly diminished the engraftment (chimerism-time AUC_0→10_ = 330.8±21 days with serum *versus* 224.6±76 days without serum, n = 3 mice/group, *P* = 0.04, one tail unpaired *t*-test) (Fig. 3, A, inset) whereas deprivation of exogenous hypoxanthine had no effect (not shown). In addition, the pathological analysis of brain, kidney, liver and spleen of HM showed vascular congestion and elevated hematocrit (70.9±11.6 in engrafted mice *vs* 46.5±0.9 in non-conditioned NOD*^scid/β2m−^* ^/*−*^ mice) as the only relevant findings (n = 6 mice·group^−1^, 8 days after starting conditioning) (Fig. 3, B). Consistently, the concentration of the circulating erythrocytes was high (9.4±1.1, ×10^9^ E·ml^−1^, n = 50 mice) (Fig. 3, C). Noteworthy, although conditioned mice showed hepatomegaly, mild splenomegaly and significant elevations in BUN, bilirubin, and total protein (Table 2), we did not observe any overt sign of disease in mice. Therefore, we could obtain a high, reproducible and well-tolerated engraftment of hE for long periods in the absence of cytoreductive therapies.

**Table 2:** Effect of repeated blood injections on clinical parameters of mice^1^.

Organ weight (gr_organ_·gr_bodyweight_·100^−1^)		Days of blood injection						
		0		7	13	21		*P* 2
Liver		3,67±1,5		*5,3±0,7*	*6,0±0,3*	*6,6±1*		<0,001
Kidney		1,1±0,04		1,2±0,12	1,09±0,1	1,2±0,14		NS^4^
Heart		0,5±0,05		0,5±0,05	0,4±0,03	0,5±0,02		NS
Lung		0,7±0,05		0,7±0,1	0,7±0,1	0,8±0,1		NS
Spleen		0,2±0,04		0,3±0,05	0,4±0,06	0,4±0,05		NS
Serum biochemical markers^3^								
ALT	IU·L^−1^	33,10±5,4	49,10±21,2		52,60±10,6		63,20±11,8	0,24
BUN	mg·dL^−1^	21,50±1	*31,30±4,9*		*34,00±2,8*		28,30±4,4	<0,001
AST	IU·L^−1^	167,80±57	161,20±29,7		192,20±26,4		226,50±50,5	0,25
TBIL	mg·dL^−1^	0,60±0	*5,30±6,4*		*1,70±0,3*		*2,80±1,2*	<0,001
TP	g·dL^−1^	4,90±0,2	*5,60±0,7*		*5,80±0,3*		5,40±0,5	0,002

1 Each experimental group was n = 5.

2 Analysis of organ weight was performed using two factor-ANOVA test followed by Bonferroni post test. Differences were considered significant if P<0,05. Values reported are the probability of the difference in weight with respect to day 0. In italics groups that differed significantly from control at day 0.

3 ALT: Alanine aminotransferase; BUN: Blood Urea Nitrogen; AST: Aspartate aminotransferase; TBIL: Total bilirrubine; TP: Total protein. Analysis of biochemical markers in serum was conducted using a general linear model multivariate contrast of means followed by a bilateral Dunnett's t test. In italics groups that differed significantly from control at day 0.

4 NS: Not significant.

### Dynamics of infection

**Figure 4:**
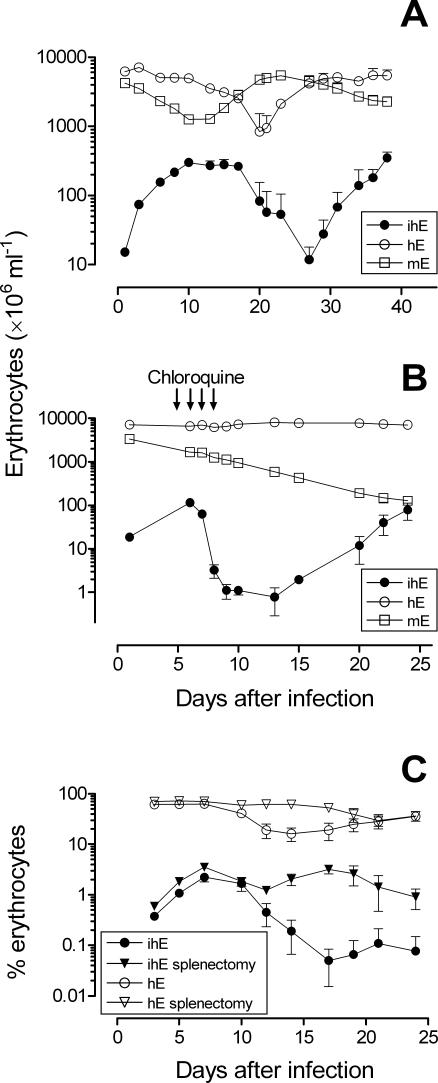
Infection dynamics in HM infected with *Pf*3D7^0087/N9^. Concentration of hE, ihE and mE in peripheral blood of infected mice. Data are the mean±SE of three mice per data point. The results are from a representative experiment out of three. (B) Abrogation of infection-induced elimination of hE by treatment with chloroquine (10 mg·Kg^−1^) and exponential growth of *Pf*3D7^0087/N9^ during recrudescence. Data are the mean concentration±SE of three mice per data point. The results are from a representative experiment out of two. (C) The effect of splenectomy on the percentage of hE and parasites in peripheral blood. The data are the means±SE of n = 6 mice·group^−1^ pooled from two independent experiments.

Next, we studied the dynamics of infection in HM infected i.v. with the established *Pf*3D7^0087/N9^ strain. We infected a group of mice (n = 3) having 40–50% circulating hE (7–10 days after starting hE injections) with 20·10^6^ *Pf*3D7^0087/N9^-infected human erythrocytes (ihE) and measured the concentration of ihE, murine erythrocytes (mE) and hE in peripheral blood of mice up to 36 days after infection. As shown in Fig. 4, A and in Table 3, the infection with *Pf*3D7^0087/N9^ followed a characteristic pattern and consistently changed the dynamics of hE and mE in peripheral blood of mice (three independent experiments, with n = 3, 4, and 3 mice, respectively). The growth of *Pf*3D7^0087/N9^ in peripheral blood of HM was associated with a severe decrease in total erythrocyte concentration with respect to uninfected HM (up to 50% of reduction) explained by the selective elimination of hE from peripheral blood of infected mice, which induced a compensatory increase in circulating mE. The treatment of infected HM with a suboptimal dose of chloroquine (10 mg·Kg^−1^, once a day for 4 days, p.o.) reduced the parasite burden and eliminated the loss of hE while the number of mE dropped continuously as in uninfected chimeric mice. Of note, recrudescent parasites grew exponentially as after initial infection. Thus, our results indicate that a high density of *P. falciparum* triggers the elimination of hE, which is associated with a severe impairment of parasite growth in which the spleen seemed to be involved. In mice, the spleen is a key organ for elimination of senescent erythrocytes [28] and ihE upon recruitment of phagocytes [26]. Interestingly, splenectomy showed negligible effects in chimerism or parasitemia during the exponential growth of the parasite, but improved chimerism (*P* = 0.032, Student's *t* test, n = 6 mice·group^−1^) and parasitemia (*P* = 0.009, Student's *t* test, non homogeneous variance, n = 6 mice·group^−1^) only when this latter reached a plateau or started to decrease (Fig. 4, C).

**Table 3:** Dynamics of infection in HM^1^.

Days after infection	% hE	% Parasitemia^2^	Parasites·ml^−1^ (×10^6^)	E·ml^−1^ (×10^6^)	hE·ml^−1^ (×10^6^)	mE·ml^−1^ (×10^6^)
1	59,7±1,5	0,1±0	15,1±2,1	10384,9±125	6193,5±110,1	4191,3±201
3	66,7±0,6	0,7±0,1	73,7±12,3	10643,9±20,1	7102,7±56,8	3541,1±65,5
6	68,5±1,5	2,1±0,1	156,2±12,3	7370,9±193,4	5050,7±152,3	2320,2±141,1
8	73,7±1,6	3,1±0,6	215,8±41,5	6875±502,4	5071,3±465,7	1803,7±77,7
10	79,7±1	4,9±0,8	299,9±37,1	6222,8±394,8	4962,6±368,2	1260,2±43,7
13	73,2±4,9	5,6±1,2	270,9±63	4821,4±351,1	3540,5±431,1	1280,9±189,8
15	62,5±7,8	5,6±1,1	278,5±73,3	4953,4±296,2	3112,3±531,9	1841,1±314,2
17	48,1±7,3	4,9±0,5	263,7±18,8	5370,3±590,4	2538,1±143,3	2832,2±731,1
20	13,3±14,6	1,3±1,5	83±101,8	5549,7±698,3	839,4±969,1	4710,4±275,7
21	15,6±10,5	0,9±1,3	57,3±80,5	5933,9±330,4	940,9±667,1	4993±563,6
23	28,6±7,3	0,8±1,2	53,2±73,4	7544,8±909,1	2115,2±460,5	5429,5±1101,4
27	46,4±16,6	0,1±0,1	11,8±8,5	8673,2±950,9	4178,2±1774,4	4495±826,4
29	54,1±11	0,3±0,3	27,5±23,3	8933,4±625,6	4905,6±1283,8	4027,8±658,9
31	59,3±10,1	0,9±0,8	67,9±60,6	8646,6±789,2	5116,2±983,2	3530,4±1018,5
34	62,3±9,3	2,1±2,2	139,2±135,6	7216,2±607,3	4519,9±948,8	2696,3±630,2
36	67,9±10,5	2,6±1,3	180,5±82,2	7815,2±1751,3	5454,5±1961,1	2360,7±605,4

1 Data are the mean±SEM of n = 3 mice infected at day 0 with 20·10^6^ *Pf*3D7^0087/N9^-parasitized erythrocytes/mouse obtained from peripheral blood of a donor mouse.

2 Total parasitemia.

Therefore, *Pf*3D7^0087/N9^ injected i.v. was able to produce sustained and reproducible infections in HM. Moreover, the growth of the parasite caused a relative anemia due to selective elimination of hE by mechanisms dependent on the density of parasites in peripheral blood.

### Characterization and validation of a standardized assay for antimalarial efficacy

**Figure 5:**
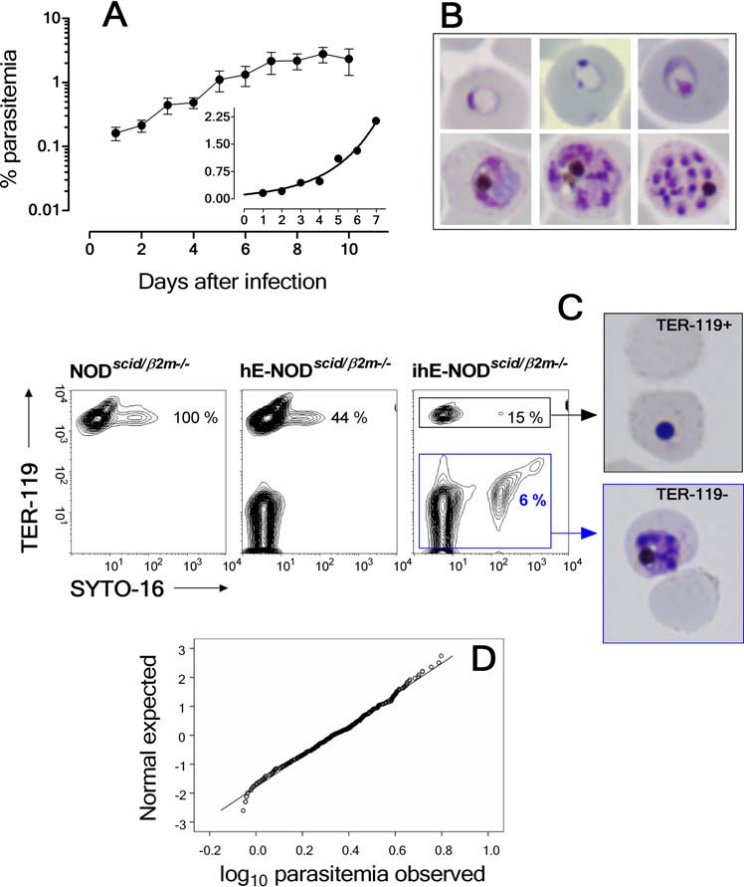
Characterization of the *P. falciparum*-malaria model for antimalarial drug testing. (A) Kinetics of parasitemia in peripheral blood of HM infected i.v. with 20·10^6^ *Pf*3D7^0087/N9^ parasites. Data are the mean±SE from 88 mice. The inset plot shows the regression line to fit an exponential growth model of the means of parasitemia of the pooled data up to day 7 after infection. (B) Giemsa-stained smears from peripheral blood of HM infected i.v. with *Pf*3D7^0087/N9^ showing the different stages of *P. falciparum*: ring (upper row on the left) to mature schizont (lower row on the right) (×1000 magnification). (C) Replication of *Pf*3D7^0087/N9^ in hE. Flow cytometry panels depict erythrocyte populations in control (left panel), uninfected HM (middle panel) and infected HM (right panel). The percentage of mE TER-119^+^ cells is indicated. *Pf*3D7^0087/N9^ (parasitemia 6%) are in TER-119^−^SYTO-16^+^ events. Pycnotic forms of *P. falciparum* were found in Giemsa stained-cytospin preparations of mE TER-119^+^ purified immunomagnetically. Viable parasites were found in purified hE TER-119^−^. Data are representative of three independent experiments. (D) Q–Q plot of normality for the variable log_10_ (parasitemia at day 7) showing observed values vs expected normal values (n = 327 mice).

We develop a standardized *in vivo* assay useful for drug discovery to demonstrate the usefulness of the murine *falciparum* malaria model. The first 10 days after infection encompassed up to five parasitic cycles of 48 h and in this period, the parasite density was low enough to allow a reproducible exponential growth of *Pf*3D7^0087/N9^ up to the maximum parasitemia. All HM infected i.v. with 20·10^6^ *Pf*3D7^0087/N9^-ihE showed a one-phase exponential association function kinetics of parasitemia up to day 7 after infection (*R* ^2^ = 0.74), with a doubling time of 1.7 days (1.5 to 1.9 days 95% Interval of confidence) (n = 88 mice) (Fig. 5, A) irrespective of AOB or Rh blood groups. Interestingly, *Pf*3D7^0087/N9^ was not strictly dependent on exogenous supplementation of hypoxanthine (parasitemia *vs* time AUC_0→10_ 17.2±4.4 days with hypoxanthine *vs* 12.1±5.1 days without hypoxanthine, n = 3 mice·group^−1^, *P = *0.15, Student's *t*-test). However, human serum deprivation impaired chimerism (chimerism *vs* time AUC_0→10_ = 669.9±88.3 days with serum *vs* 289.7±152 days without serum, *P = *0.02, Student's *t*-test) and parasitemia (parasitemia *vs* time AUC_0→10_ = 12.7±0.1 days with serum *vs* 0.6±0.9 days without serum, n = 3 mice·group^−1^, *P = *0.002, Student's *t*-test).

During the assay period, all the erythrocytic stages of the parasite, except gametocytes, which *P. falciparum* 3D7 does not produce neither spontaneously *in vitro* nor in myelodepleted HM [11], were noticeable (Fig. 5, B). Multiparasitized erythrocytes were rare and productive infections occurred apparently only in hE (Fig. 5, C), as described *in vitro* [29]. Parasites grew asynchronously and sequestration of mature stages was not apparent in mice. Consistently, we did not see endothelial inflammation, sequestration, or margination of parasites in brain, kidney, liver or spleen studied at day 8 after infection. In addition, we did not find intravascular hemolysis, intravascular coagulation or major histopathological changes in those organs. However, infection of HM with *Pf*3D7^0087/N9^ decreased vascular congestion and increased the number of leukocytes in the spleen (Fig. 3, B).

**Figure 6:**
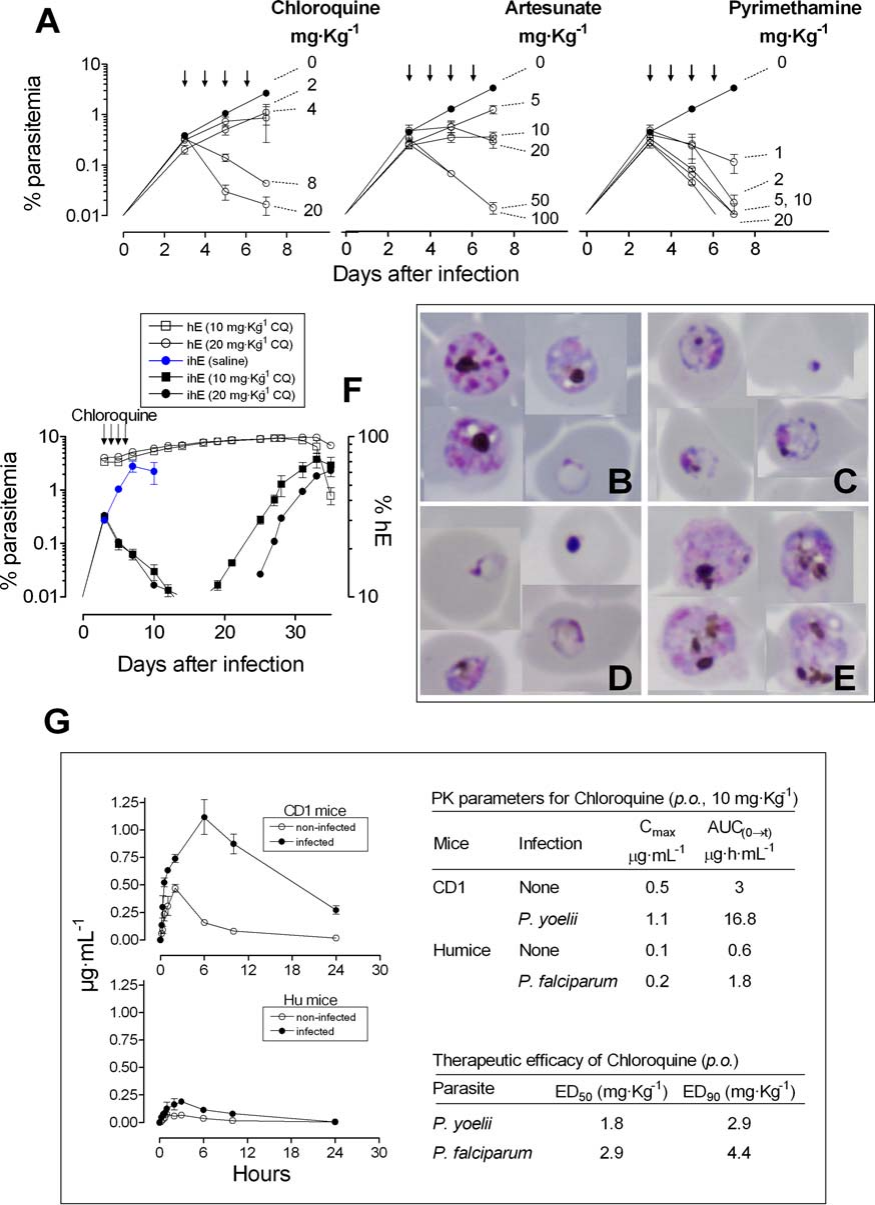
Validation of the *P. falciparum* 4-day test. (A) Therapeutic efficacy of chloroquine, artesunate, and pyrimethamine using the standard *P. falciparum* 4-day test. Data are the mean parasitemia±SE of n = 3 mice·group^−1^ from a single experiment using 45 mice. (B, C, D, E) Representative blood smears 48 h after starting treatment with saline, chloroquine (20 mg·Kg^−1^), artesunate (25 mg·Kg^−1^) or pyrimethamine (20 mg·Kg^−1^), respectively. The remaining parasites in peripheral blood after treatment with chloroquine or artesunate were pycnotic cells and disrupted trophozoites. Pyrimethamine led to swollen late trophozoites with prominent granules of hemozoin (×1000 magnification). (F) Logarithmic growth of *Pf*3D7^0087/N9^ during recrudescence after treatment with chloroquine (10 and 20 mg·Kg^−1^, p.o., u.i.d.) in a standard 4-day test. Data are the mean parasitemia±SE of three mice/group. (G) Exposure-therapeutic efficacy relationships of chloroquine in the *P. yoelii* or the *P. falciparum* murine models of malaria. Data are the mean concentration of chloroquine in blood of n = 3 mice·group^−1^.

Next, we set up a standard assay of efficacy (Materials and Methods) and validated the assay using three established antimalarials: chloroquine, pyrimethamine and artesunate. These antimalarials were active *in vitro* against *P. falciparum* 3D7 and the *in vivo* strain *Pf*3D7^0087/N9^ (see Table 1). All three compounds were active when administered orally once a day for 4 consecutive days, with a clear dose-response effect, (Fig. 6, A). The 95% intervals of confidence (IC 95%) for the 90% effective dose (ED_90_) of these antimalarials were 2.5–6.3, 0.5–1.3, and 10.2–15.2 mg·Kg^−1^, for chloroquine, pyrimethamine and artesunate, respectively. Only with dosages that engendered quantitative antiparasitic effects, circulating parasites showed characteristic structural alterations (Fig. 6, B–E). Interestingly, after treatment with chloroquine, recrudescence curves seemed to show a dose-effect relationship (Fig. 6, F). We also compared the efficacy and pharmacokinetics of chloroquine in a standard murine model (*P. yoelii* infection in CD1 mice) and the *P. falciparum* model. The exposure of chloroquine in whole blood of non-infected controls or *P. yoelii*-infected CD1 mice was 4–5 times higher than in uninfected or infected humanized NOD*^scid/β2m−/−^* mice (Fig. 6, G). However, the pharmacokinetic profiles were identical in both murine systems. Of note, infection increased the levels of chloroquine detected in blood of infected mice in both cases. This effect is likely due to accumulation of the drug in infected erythrocytes [30]. Consistently, the doses required to achieve equivalent therapeutic efficacy were slightly higher in the *P. falciparum* model compared to *P. yoelii* model (Fig. 6, G).

**Figure 7:**
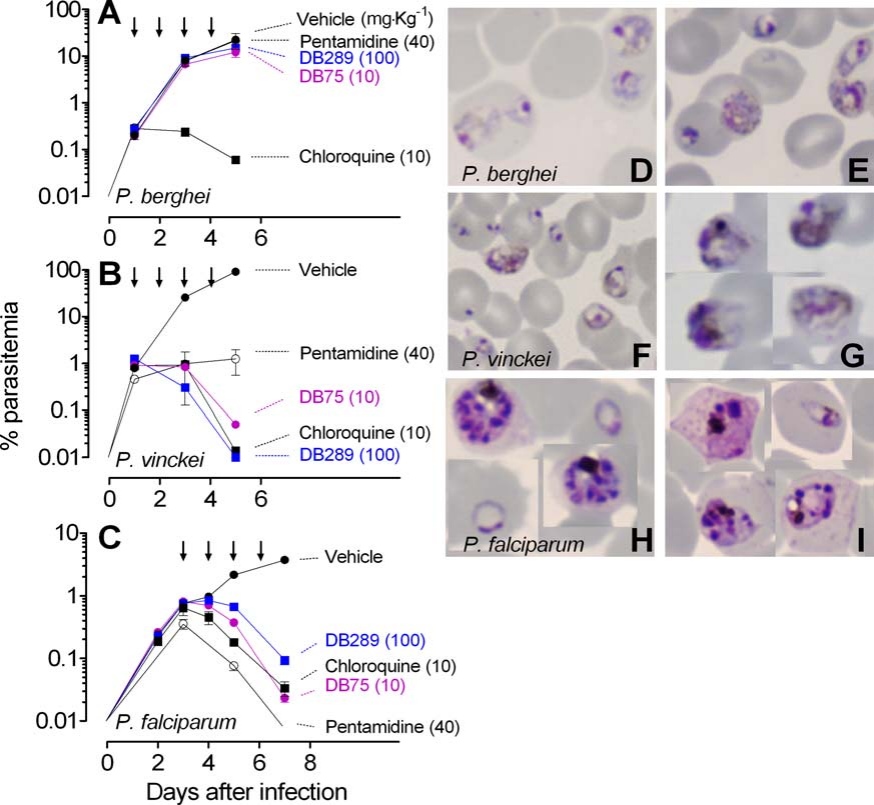
Therapeutic efficacy of diamidine derivatives against *P. berghei*, *P. vinckei* and *P. falciparum*. (A, B, C) Therapeutic efficacy of pentamidine (40 mg·Kg^−1^, u.i.d., s.c.), DB75 (10 mg·Kg^−1^, u.i.d., s.c.) or DB289 (100 mg·Kg^-1^, u.i.d., p.o.) against *P. berghei*, *P. vinckei* and *P. falciparum*, respectively. We started treatment when parasitemias where comparable and administered compounds for 4 days. (D, E) Giemsa-stained blood smears from mice infected with *P. berghei* obtained 48 h after starting treatment with vehicle or DB75 at 10 mg·Kg^−1^, respectively (×1000 magnification). Neither relevant cellular damage nor significant inhibition of parasitemia were observed at the time of sampling. (F, G) Giemsa-stained blood smears from mice infected with *P. vinckei* obtained 48 h after starting treatment with vehicle or DB75 at 10 mg·Kg^−1^, respectively (×1000 magnification). Parasites from treated mice were mostly abnormal late trophozoites. (H, I) Giemsa-stained blood smears from mice infected with *P. falciparum* obtained 48 h after starting treatment with vehicle or DB75 at 10 mg·Kg^−1^, respectively (×1000 magnification). Parasites from treated mice were mostly abnormal late trophozoites.

To further validate the assay, we also tested the diamidine analogs of pentamidine DB75 and DB289, its orally bioavailable pro-drug. These are promising antimalarial compounds that have proved to be effective in patients infected with *P. vivax* or suffering acute uncomplicated *P. falciparum* malaria [31]. Interestingly, diamidines seem to require parasite-induced permeability pathways in infected erythrocytes [32] and might have several intracellular targets [31]. Pentamidine has been shown to be almost inactive against *P. berghei* but very effective against *P. vinckei in vivo* [33]–[35]. Our results indicated that pentamidine (40 mg·Kg^−1^, u.i.d., s.c.), DB75 (10 mg·Kg^−1^, u.i.d., s.c.) and DB289 (100 mg·Kg^−1^, u.i.d., p.o.) were inactive against *P. berghei* (Fig. 7, A) but showed a marked activity against *P. vinckei* (Fig. 7, B). Interestingly, pentamidine (40 mg·Kg^−1^, u.i.d., s.c.), DB75 (10 mg·Kg^−1^, u.i.d., s.c.) and DB289 (100 mg·Kg^−1^, u.i.d, p.o.) were also effective against *P. falciparum* in the murine 4-day test (Fig. 7, C). This indicated that DB289 was metabolized to active compounds (presumably DB75) in humanized mice because DB289 is inactive *in vitro* and depends on hepatic metabolism to exert activity *in vivo* [31]. Consistently, we did not found dramatic changes in the morphology of *P. berghei* in blood smears taken from infected mice 48 h after starting treatment with compounds, (Fig. 7, D and E). However, the remaining parasites in peripheral blood of *P. vinckei*- (Fig. 7, F and G) and *P. falciparum*-infected mice (Fig. 7, H and I) were abnormal late trophozoites-early schizonts, suggesting that the transition to schizont stage might be an especially sensitive step to diamidines. These results strongly suggested that there were significant differences between *P. falciparum* and *P. berghei* in their response to diamidine derivatives, either because of differences in cellular targets and/or the specificity of the mechanisms of transport of diamidines into hE. Thus, according to our results, *P. vinckei* would be a better surrogate of *P. falciparum* than *P. berghei* for testing *in vivo* diamidine derivatives. These results suggested that the *falciparum* murine model of malaria might be used to select surrogate standard rodent models that had better correlation with *P. falciparum* when testing *in vivo* new families of compounds.

## Discussion

We describe a reproducible murine model of *P. falciparum* infection by generating *in vivo* parasite strains able to grow reproducibly in HM and demonstrate its value implementing a tool for drug discovery of new antimalarials.

We succeeded implementing a reproducible infection with *P. falciparum* using the *in vivo* experimental procedure described by Moore et al [12]. However, these authors failed to establish a model of infection useful for drug discovery. Several reasons might explain this discrepancy. First, the selection of the murine background for initial infections with *in vitro*-cultured parasites. In our hands NOD*^scid/β2m−/−^* but not NOD*^scid^* showed productive infections after i.p. infection with *in vitro* growing *P. falciparum* 3D7. The deficiency in β2-microglobulin impairs NK cell activity [23] and leads to iron overload in the liver [23], [36]. Thus, additional defects in the innate immune system of NOD*^scid/β2m−/−^*, likely related to defective activation of phagocytes, may lower the initial selective pressure on *P. falciparum*. Secondly, the daily injection of human serum and hypoxanthine might facilitate soluble factors supporting, directly or indirectly, the growth of *P. falciparum* [16] and the engraftment of hE in mice [7]. Thirdly, the variants of *P. falciparum* that we selected *in vivo* had to be competent to overcome the innate immune system of mice whereas the adaptation of the parasite to mouse serum *in vitro* did not establish that selection pressure [12]. Consistently, the *in vivo* selection passages led to variants of *P. falciparum* 3D7 carrying potential rearrangements to variable molecules encoding antigenic determinants, which is a well known mechanism to evade the immune system [37]. In fact, an improved ability to skip phagocytes might explain the lack of synchronization *in vivo* of *Pf*3D7^0087/N9^. Hopefully, using *Pf*3D7^0087/N5^, *Pf*3D7^0087/N9^, *Pf*V1/S^0176/N7^ and *Pf*V1/S^0176/N10^ and other strains in development for new drug discovery programs, we should be able to map the genomic rearrangements and compare intra- and inter-strain genotypic and phenotypic changes associated to the process of *in vivo* selection in HM.

The daily injection of hE caused the substitution of mE by hE and polycythemia (hematocrit up to 90%) in mice. As the clearance of erythrocytes in mice seems to be dependent on the age of erythrocytes and random mechanisms [38], it is likely that the decrease of mE reflects a non-selective process of clearing erythrocytes in excess. HM were able to compensate polycythemia and the erythrocyte concentration was always below 12·10^9^ E·ml^−1^. Likely, the mechanisms of adaptation to polycythemia are similar to those found in polycythemic transgenic mice overexpressing human erythropoietin [39], which include vasodilatation, higher flexibility and mean corpuscular volume in newly generated reticulocytes, accelerated aging of erythrocytes and enhanced phagocytosis. The relevance of polycythemia in the *falciparum* murine model remains to be addressed. However, it is conceivable that the saturation of the homeostatic mechanisms controlling erythrocyte numbers may minimize hE rejection and might be accountable for the high reproducibility of the kinetics of parasitemia compared to models not showing this characteristic [8], [9], [11]. Importantly, despite the high hematocrit achieved in HM, we have not detected deaths related to toxicity in more than one thousand mice used in regular efficacy assays. In contrast, the most recent version of phagocyte-depleted HM reported a 25% rate of deaths in HM not infected with *P. falciparum* even though hematocrit measurements were almost normal (36 to 52%) [11], a fact that points to toxicity of the phagocyte depletion method.

Parasite density in peripheral blood is a key driver of the model dynamics. After i.v. infection, *Pf*3D7^0087/N9^ causes a selective elimination of hE, but as long as the parasite density is below a threshold, it does not alter significantly the dynamics of the system and *Pf*3D7^0087/N9^ grows exponentially. Noteworthy, the selective clearance of uninfected hE might have some clinical relevance because anemia is the most frequent clinical complication of *falciparum* malaria [40] and *P. falciparum*-induced elimination of hE is a key factor in its pathogenesis [41], [42]. Infection-induced membrane rigidification [43] and selective tagging with merozoite RSP-2 rhoptry protein of hE [44] have been proposed as relevant mechanisms, and could be involved in our model as well. In agreement with previous reports [8], [9], [11], our results suggest that the recruitment and activation of phagocytes have a central role limiting the growth of *P. falciparum* in HM. Thus, we anticipate that the overall dynamics of the system described in this paper can be manipulated by deleting *in vivo* activation and/or homing receptors of phagocytes. Moreover, it is important to note that this dynamic of infection differs substantially from the model proposed by Moreno et al. in phagocyte-depleted HM after intraperitoneal infection [26]. In this model, only depletion of phagocytes would allow the intraperitoneal growth of *P. falciparum*. Ultimately, the intraperitoneal infection of phagocyte-depleted HM with *P. falciparum* would lead to spontaneous parasite clearance or mouse death depending on the capacity of surviving peritoneal phagocytes to control parasite replication in the peritoneum. Consistently, no successful intravenous infection have been described in phagocyte-depleted HM models.

Willimann et al [45] showed that *P. falciparum* ItG2.F6-infected erythrocytes injected in *scid* mice bind selectively *in vivo* to ICAM-1-positive murine endothelium. Whether this phenomenon takes place in HM infected with *P. falciparum* remains to be addressed. However, we found all stages of the parasite (except gametocytes) circulating in peripheral blood, without microscopic evidence of attachment or manifest clinical symptoms in HM. Thus, our results suggested that there was no prominent adhesion of the strains generated in HM to murine endothelium *in vivo.* The *in vivo* selection procedure employed to generate *P. falciparum* strains might favor the expansion of parasites with reduced ability to bind murine endothelium. However, it is important to note that Moreno et al [11] did not find margination in phagocyte-depleted HM infected with *P. falciparum* even though no previous selection or adaptation of the parasite was employed. In any case, the lack of adhesion was an advantage for the evaluation of antimalarial drugs since the effect of the drugs on all stages is readily observable and the evaluation of the dose/exposure-response relationships is easier and technically more accurate. Conversely, this may be the most important limitation of the *falciparum* model described here for pathology or cytoadhesion studies. It is interesting to note that Moore et al [12] found that *P. falciparum* 3D7 adapted *in vitro* to murine ascites could differentiate to gametocytes upon infection of HM. This is in contrast with the results reported in this paper using *Pf*3D7^0087/N9^ and by Moreno et al [11] in phagocyte-depleted HM using *P. falciparum* 3D7. As the three approaches have substantial differences, there are no obvious explanations for this discrepancy. *P. falciparum* 3D7 does not produce spontaneously gametocytes *in vitro*. Thus, it would be likely necessary to test other strains, particularly clinical isolates, in the *in vivo* selection procedure in HM to assess the capacity of the *falciparum* HM to allow the differentiation to gametocytes, a trait already proven for phagocyte-depleted HM [11]. In any case, it is unlikely that any *falciparum* murine model can reflect all the pathophysiological characteristics of human *falciparum* malaria. Thus, devising specialized *falciparum* murine models that modeled reproducibly key aspect of the parasite's biology in humans might be a more realistic approach.

Discovery of new antimalarial drugs is a priority for global health programs. With the exception of humans, the murine *falciparum* models are the only ones that enable the evaluation of antimalarial drugs against *P. falciparum* inside human erythrocytes *in vivo*. Thus, they may be unique tools to study the effect of drug exposure in blood on parasitemia. Moreno et al. [9], using the sensitive strain NF54 and the chloroquine/quinine resistant T24 strain of *P. falciparum* demonstrated that using phagocyte-depleted HM having at least 6 days of stable parasitemia it was possible to detect active compounds against *P. falciparum.* However they did not show whether their experimental design could address dose-response relationship for any of the standard antimalarials used [9]. To the best of our knowledge, our paper describes the first assay in which a dose-response relationship for antimalarials has been reported in an antimalarial assay set up using a *falciparum* murine model. Thus, our model enabled us to compare the relative potency of compounds *in vivo*, assessing the actual blood exposures of each compound with its therapeutic efficacy in the same animal species, using a highly reproducible standardized assay, and avoiding potentially interfering toxic reagents. Indeed, the antimalarial assay described has been used to support the lead optimization of a number of antimalarial projects funded by Medicines for Malaria Venture, including 4-(1)-pyridones, falcipain inhibitors, dihydrofolate reductase inhibitors, and diamidine derivatives (manuscript in preparation). It is worth mentioning that the actual exposures in blood of several compounds tested were lower in HM than in normal CD1 mice in all the cases studied. Likely, an increase in vascular volume due to vasodilatation contributed to lower exposures. However, changes in absorption and metabolism might play an important role, either increasing or decreasing the actual blood exposures of new families of compounds. Considering its use in drug discovery, the model described in this paper is especially well suited to evaluate compounds inhibiting *P. falciparum*-specific targets at any stage of the drug discovery process. However, it is more expensive than the standard rodent models of malaria, which are reliable, reproducible and affordable. Hence, it seems logical that these standard models should be the first choice for preliminary experiments of therapeutic efficacy if they proved to be good surrogates of *P. falciparum*. To test this point, the *falciparum* model described in this paper might provide a reference to select appropriate rodent plasmodial species to test *in vivo* new families of antimalarials, and, given their urgent need, to re-evaluate promising compounds active *in vitro* against *P. falciparum* that were discarded because they failed *in vivo* against rodent *Plasmodium spp*.

In this paper, we demonstrate that it is possible to generate *in vivo* strains of *P. falciparum* competent to grow reproducibly in peripheral blood of HM without using toxic treatments to deplete phagocytes. These parasite strains are tools that may be as useful in malaria as the strains adapted to *in vitro* culture, currently the cornerstone of research in *P. falciparum*. As the use of competent strains guarantees a reliable growth of the parasite in HM, it should be possible to devise new *falciparum* murine models using humanized immunodeficient mice with specific defects in phagocyte activity/activation and expressing human adhesion molecules in endothelium. These tools will hopefully open up more ways to study the biology of the erythrocytic stages of *P. falciparum in vivo* and its molecular responses to selective pressures imposed by antimalarials or the human immune system reconstituted in mice [26], [46], [47].

## Materials and Methods

### Compounds

Chloroquine, artesunate, artemisinin, pentamidine and pyrimethamine were purchased from Sigma (St. Louis, MO). Atovaquone is owned by GlaxoSmithKline. DB75 and DB289 were obtained from Medicines for Malaria Venture. Chloroquine diphosphate was dissolved in saline. Artesunate (AAPIN Chemicals Ltd., Abingdon, UK) was prepared as suspensions in water 1% hydroxipropil-β-cyclodextrine (FLUKA, Seelze, Germany). Pyrimethamine was prepared as suspensions in water 1% methylcellulose (Sigma). Pentamidine, DB75 and DB289 were dissolved in 70% Tween 80 and 30% ethanol and then further diluted 1/10 with distilled water. All compounds were dissolved in RPMI 1640 human serum 10% DMSO 0.2% for *in vitro* testing.

### Parasites

Drs. E. Dei-Cas and L. Delhaes from Institut Pasteur (Lille, France) kindly donated uncloned *P. falciparum* 3D7, *Plasmodium berghei* and *Plasmodium yoelii* 17 X. Both *Plasmodium falciparum* V1/S and *Plasmodium vinckei* were obtained from the Malaria Research and Reference Reagent Resource Center (ATCC, Manassas, VA, USA). Parasites were cryopreserved and thawed using the glycerol/sorbitol method with minor modifications [48], [49].

### Mice

Female CB17*^scid^* (CB17/Icr.Cg-Prkdc*^scid^*), CB17*^scid/beige^* (CB17/Icr.Cg-Prkdc*^scid^* Lyst*^bg^*/Crl), NIH-III*^beige/xid/nude^* (Crl:NIH-*Lyst^bg^ Foxn1^nu^ Prkdc^xid^*), NOD*^scid/β2m−/−^* (NOD.Cg-*Prkdc^scid^B2m^tm1Unc^*/J) and NOD*^scid^* (NOD.CB17-*Prkdc^scid^*/J) were purchased from The Jackson Laboratory (Bar Harbor, ME). A colony of NOD*^scid/β2m−/−^* mice was raised and maintained by Charles River Laboratories (L'Arbresle, France). The mice were used at 8 weeks of age. The mice were fed with autoclaved tap water and γ-irradiated pelleted diet *ad libitum*. All the experiments were approved by DDW-Ethical Committee on Animal Research and were conducted according to European Union legislation and GlaxoSmithKline policy on the care and use of animals.

### Blood

We employed incomplete donations or erythrocyte concentrates of malaria-negative donors, generously provided by the Spanish Red Cross blood bank in Madrid, Spain. Before injection, blood stored at 4°C was washed twice with RPMI 1640, 25 mM HEPES (Sigma) containing 7·10^−3^ mM hypoxanthine (Sigma) at room temperature. The buffy coat was removed by aspiration (if required) and erythrocytes resuspended at 50% hematocrit in RPMI 1640, 25% decomplemented human serum (Sigma) 3.1 mM hypoxanthine. Finally, the blood suspension was warmed at 37°C for 20 minutes before intraperitoneal injection with 1 ml of hE suspension every day during the experiments.

### *In vitro* cultures

Asexual stage *P. falciparum* 3D7 parasites were maintained in continuous culture as described [50] with minor modifications. Growth inhibition assays were assessed by monitoring [^3^H]-hypoxanthine uptake as described previously [51]. Inhibitory concentrations at 50 and 90% (IC_50_ and IC_90_, respectively) were determined by fitting a sigmoid dose-response curve to experimental data using GraphPad Prism 4.0 (GraphPad Software, San Diego, CA).

### Parasite typing

Polymorphic microsatellite fingerprinting of *Plasmodium falciparum* within a multicopy *rif* repetitive element [52] (*Pf*RRM) was carried out following the method published by Su et al. [27] with minimal modifications. Genomic DNA was PCR amplified using the described primers set (Fwd TACGTTACATTATGTTTTA labeled with 6-FAM and Rev ATATGTATTGCGCTTTTA). The size of the amplicons was analyzed in an automated single-capillary genetic analyzer Abi Prism 310 Genetic Analyzer (GMI, Inc., Ramsey, MN).

### Histology

Female NOD*^scid/β2m−/−^* mice were injected daily with 1 ml of hE for 10 days and infected or not (conditioned controls) with 20·10^6^ *P. falciparum*-infected erythrocytes. Brain, lungs, kidneys, liver and spleen from all experimental groups were aseptically removed at day 8 after infection and fixed *in toto* by immersion in neutral buffered 10% formalin solution for 48 h. Serial cross sections of the organs were obtained and fixed again in formalin for 12 h. Finally, tissue sections were embedded in paraffin, 5 µm thick sections were prepared and these were stained with hematoxylin and eosin and examined. Blood smears for assessing parasites were performed as described [53].

### Flow cytometry

All mAb were from BD Biosciences Pharmingen (San Diego, CA). We measured the expression of murine myeloid markers Ly-6G and F/480 in leukocytes exactly as described [54]. Measurement of parasitemia in peripheral blood was assessed by measuring the percentage of hE containing nucleic acids. Blood samples were taken from the tail lateral vein (2 µL), collected onto 100 µL of saline containing 10 µg ml^−1^ PE-conjugated rat IgG2b anti-mouse erythrocyte TER-119 mAb [55], incubated for 20 min, fixed with glutaraldehyde and stained with YOYO-1 (Molecular Probes, Leiden, The Netherlands) as described previously [56]. Engraftment with hE was routinely measured as the percentage of TER-119^−^ erythrocytes, which rendered the same results obtained by measuring glutaraldehyde-fixed erythrocytes with PE-conjugated rat anti-human glycophorin A mAb. For measurement of the concentration of cells in blood, 2 µL of blood were obtained from the tail lateral vein, collected onto 100 µL of staining solution (SYTO-16 (Molecular Probes) at 5 µM [57], [58] and 10 µg ml^−1^ PE conjugated anti-mouse TER-119 monoclonal antibody [55]) in 96-well V-bottomed plates, incubated for 20 minutes at room temperature and neutralized for 10 minutes with 10 µL of 0.25% glutaraldehyde per well. For acquisition, 30 µL of the sample were added to TruCounts™ tubes (Becton Dickinson) [59] containing a known number of lyophilized fluorescent beads suspended in 300 µL of saline. The sensitivity to detect infected erythrocytes was 0.01% and measurements were linear across the range of sensitivity. Either YOYO-1 or SYTO-16 were used because they showed no significant differences for parasitemia determinations (Pearson's coefficient of correlation r = 0.998) (Jiménez-Díaz et al, submitted).

### Magnetic sorting of erythrocytes

Purification of TER-119^+^ erythrocytes was performed exactly as described previously [56]. Briefly, 10 µL of blood were washed with PBS, resuspended in 0.25 ml of PBS 1% FCS (Sigma) and stained with biotinylated rat anti-mouse TER-119 (BD Biosciences Pharmingen) at 10 µg ml^−1^ for 30 min at room temperature. After washing twice with separation buffer (PBS 0.5% BSA 2 mM EDTA), the cells were resuspended in 0.45 ml of this buffer plus 50 µL of streptavidin conjugated BD iMag DM particles (BD Biosciences Pharmingen) and incubated for 30 min at 4°C. Finally, 0.55 ml of separation buffer was added to the sample and the cellular suspension was exposed to DYNAL MPC-1 Magnetic Particle Concentrator (Dynal, Oslo, Norway) for 6 min. Negative and positive fractions were carefully collected and subjected to another cycle of purification. The quality of purification was assessed by flow cytometry as described [56].

### Evaluation of *in vivo* antimalarial therapeutic efficacy

We measured the efficacy of antimalarial compounds against *P. yoelii* or *P. berghei* in a ‘4-day test’ [60] as described previously [56]. We adapted this assay to measure the therapeutic efficacy against *P. falciparum*. Cohorts of age matched female (40 to 80, depending on the experiment) NOD*^scid/β2m−/−^* were injected i.p. daily with hE throughout the experiment. When the mice reached ≥40% of chimerism in peripheral blood (7–9 days after initiation of injections), we infected them i.v. with 20·10^6^ parasites obtained from infected donors and the mice were randomly distributed in groups of n = 3 mice·group^−1^ (day 0). Treatments were administered from day 3 until day 6 after infection. We measured the percentage of TER-119^−^YOYO-1^+^ (or SYTO-16^+^) hE in peripheral blood at day 7 after infection and recrudescence up to day 35 if the parasitemias were below our detection limit (0.01%). We determined the minimum size of each experimental group (n = 3) to detect a reduction of 50% in parasitemia in peripheral blood assuming Type I error α = 0.05 (confidence level) and Type II error β = 0.2 (power of the assay). This sample size was calculated upon the distribution of the decimal logarithm of the mean percentage of TER-119^−^YOYO-1^+^ parasitized hE, which is normally distributed (log parasitemia 0.334±0.01; n = 327 mice, *P*>0.2, Kolmogorov–Smirnov test of normality with Lilliefors' correction of significance) (Fig. 5D). The therapeutic efficacy of compounds was expressed as the effective dose (mg·Kg _bodyweight_^−1^) that reduces parasitemia by 90% with respect to vehicle treated groups (ED_90_). All compounds and corresponding vehicles were administered orally at 20 ml·Kg^−1^ or subcutaneously at 10 ml·Kg^−1^, as appropriate. Chloroquine is included as quality control for each *in vivo* assay.

### *In vivo* pharmacokinetic studies in mice

Experimental mice (n = 3 mice·group^−1^) received a single bolus dose of chloroquine (10 mg·Kg^−1^, p.o. in sterile saline). Blood samples (25 µl) were collected by puncture of the lateral tail vein, mixed 1∶1 with de-ionized water 0.1% saponin and stored frozen at −70°C until use. After protein precipitation and liquid/liquid extraction, the samples were assayed by LC/MS using ESI in Q1 M+1 mode conditions by selected ion monitoring in an API 2000 mass spectrometer (Applied Biosystems Sciex, Foster City, CA) coupled to a HPLC chromatograph (Agilent HP1100 Series, Agilent Technologies Spain). Quantification was conducted by comparison to calibration curves. Blood concentrations *versus* time data were analyzed by non-compartmental analysis (NCA) methods using WinNonlin® Professional Version 4.1 (Pharsight Corporation, Mountain View, CA) and GraphPad Prism 4.0 (GraphPad Software).

### Clinical evaluation of mice

The clinical evaluation of mice was performed through a functional observational battery in order to assess behavior and nervous system responses [61]. Blood samples were taken in heparin-Litium tubes (Sarstedt, Nümbrecht, Germany), centrifuged at 1500 g for 10 minutes and plasma was separated to be analyzed in a Beckman CX5 biochemical autoanalyzer (Beckman Coulter, Fullerton, CA). Hematology analyses were performed using a Coulter AcT.5.Diff (Beckman Coulter).

### Statistics

Observational studies were performed with pooled data from mice used in routine standard *in vivo* assays of new antimalarials. Sample sizes for comparative experiments were calculated to detect a decrease of 50% in the mean of control group for Type I error α = 0.05 and 80% of power, unless otherwise stated. For comparison of kinetic curves we assessed the area under the curve (AUC) instead of comparing individual data points that do not account for the temporal dimension of data [62].The variables percentage of chimerism in peripheral blood of mice and percentage of parasitemia vs time AUC_0→t_ were normally distributed. Comparison of the mean of each experimental group was analyzed by Student's *t* test, one factor ANOVA followed by Dunnett's, Tukey's HSD or Games-Howell post tests, two factor ANOVA followed by Bonferroni post-test or Multivariate General Linear Model followed by a Dunnett's post test, as appropriate. Homogeneity of variances was assessed by Levene's test. Data variability is expressed as standard error (SE) throughout the paper. Analysis was performed using SPSS 13.0 for Windows (SPSS Inc., Chicago, IL). Probability values larger than 0.05 were considered not significant.
